# A multi-dimensional comprehensive evaluation of the nutritional value for four woody plants in desert steppe

**DOI:** 10.3389/fpls.2026.1912885

**Published:** 2026-07-29

**Authors:** Jun Zhang, Meiru Hou, Siqi Dong, Juhong Liu

**Affiliations:** College of Science, Inner Mongolia Agricultural University, Hohhot, China

**Keywords:** comprehensive evaluation, desert steppe, discrimination test, nutritional value, stem-leaf

## Abstract

This study evaluated the nutritional value of four dominant woody species (*Artemisia frigida*, *Bassia prostrata*, *Krascheninnikovia ceratoides*, and *Caragana microphylla*) in the desert steppe of Siziwang Banner, Inner Mongolia. Sample plots (1 m × 1 m) were randomly established, with three subsamples collected per plot as technical replicates in mid-to-late August, with leaves and stems manually separated for measurement of crude protein (CP), ether extract (EE), crude ash (CA), neutral detergent fiber (NDF), and acid detergent fiber (ADF). Analysis of variance (ANOVA) was conducted across three dimensions (stem vs. leaf, same part across species, and whole plant), followed by CRITIC-GRA-TOPSIS comprehensive ranking and discrimination testing for robustness validation. The results revealed significant nutritional differences between stems and leaves, with leaves consistently exhibiting higher CP and lower fiber than stems—confirming that whole-plant analysis masks substantial component-level variation. *Krascheninnikovia ceratoides* leaves achieved the highest CP (30.05%), approaching high-quality leguminous forages, while stem CP values across all species fell below maintenance requirements for grazing ruminants. Based on comprehensive rankings, *Krascheninnikovia ceratoides* ranked first in leaf and whole-plant dimensions, followed by *Bassia prostrata*; *Bassia prostrata* ranked first in stem quality. *Artemisia frigida*, showed the lowest whole-plant nutritional value. These findings support prioritizing *Krascheninnikovia ceratoides* and *Bassia prostrata* for conservation and reseeding in degraded pastures, while *Artemisia frigida*, is best retained as an ecological barrier species rather than a primary forage source.

## 1 Introduction

Desert steppes account for one-quarter of China’s total grassland area and represent the last resort for winter grazing in Inner Mongolia’s pastoral regions. In this ecologically fragile region, woody plants constitute 40–60% of the plant community and serve as the dominant vegetation type (Teleki et al., 2020). The nutritional quality of these woody plants directly determines winter supplementary feeding levels for livestock and influences ecosystem service value. However, the traditional “whole-plant sampling–laboratory routine analysis” approach overlooks significant differences in nutrient content and digestibility between shrub leaves and stems (Beaty and Engel, 1980), leading to a disconnect between evaluation results and the actual plant parts consumed by livestock. Therefore, establishing a multidimensional nutritional evaluation system based on plant components (stems vs. leaves) is of critical scientific and practical importance for accurately assessing desert steppe forage resources and optimizing grazing regimes.

Substantial research has demonstrated that forage nutritional value varies significantly among species (Faji et al., 2021; Ramos et al., 2022; Salazar et al., 2024), across seasons (Dalle, 2020), and among growth stages (Kara, 2021). Critically, leaves generally contain higher crude protein (CP), energy, and mineral content than stems, and forage with a higher leaf-to-stem ratio exhibits superior nutritional value (Mgang et al., 2021). Despite this well-established pattern, most studies continue to treat the “whole plant” as the unit of analysis, overlooking the substantial differences in cell wall content, secondary metabolites, and digestibility between leaves and stems. Research that simultaneously evaluates multiple nutritional dimensions across different plant components remains insufficient.

In terms of evaluation methodology, international feed nutrition assessment has shifted from a single-indicator (e.g., CP) approach to a multi-dimensional framework encompassing energy, digestibility, and intake (Sauvant and Noziere, 2016). The reliance on single indicators is insufficient to reflect the complex, synergistic nutritional characteristics of forages; thus, multi-dimensional comprehensive evaluation has become an inevitable trend. Various comprehensive evaluation methods have been applied to forage nutritional assessment, including Analytic Hierarchy Process (AHP) (Mukherjee et al., 2018), Principal Component Analysis (PCA) (de Carvalho et al., 2022; Sayed et al., 2022), Entropy Weighting (EW) (Guo et al., 2022), and Cluster Analysis (Bendia et al., 2021). However, each of these methods has inherent limitations: AHP relies on subjective judgments, PCA captures only linear relationships, EW neglects indicator interrelationships, and Cluster Analysis requires subjective interpretation.

The Technique for Order Preference by Similarity to Ideal Solution (TOPSIS) is a classic multi-attribute decision-making approach that ranks alternatives based on their distances from both the positive and negative ideal solutions (Thakkar, 2021). It has been successfully applied to evaluate forage yield and quality (Zhang et al., 2018; Duan et al., 2024). Grey Relational Analysis (GRA) determines correlation by comparing geometric similarity between data sequences, offering advantages in analyzing complex systems with limited samples (Pakkar, 2016; Chi et al., 2022; Chen et al., 2023). The CRITIC weighting method objectively determines weights by simultaneously considering the comparative strength of indicators and the conflicts between them (Diakoulaki et al., 1995), offering a more comprehensive and objective weight allocation than entropy weighting alone.

Traditional TOPSIS only calculates the Euclidean geometric distance between alternatives and the ideal solution in space, focusing on static ranking that merely reflects the proximity of “position”. When two alternatives are very close in distance to the positive or negative ideal solution, it becomes difficult to effectively distinguish their relative merits, potentially leading to unreliable ranking results. In contrast, GRA analyzes the shape similarity and trend consistency among data sequences, placing greater emphasis on dynamic correlations. Therefore, by integrating both approaches and applying the CRITIC weighting method, the CRITIC-GRA-TOPSIS comprehensive evaluation method is established. This method objectively weights nutritional indicators, achieving unified analysis of static structure and dynamic processes. It can measure both the numerical proximity of alternatives to the ideal solution and the consistency of their trend changes, resulting in a more comprehensive evaluation dimension.

Accordingly, this study selected the native desert steppe of Siziwang Banner, Ulanqab City, Inner Mongolia as the research area. Four dominant woody species in this region-*Artemisia frigida*, *Bassia prostrata*, *Krascheninnikovia ceratoides*, and *Caragana microphylla* which collectively account for over 70% of the local woody plant biomass and serve as the primary winter forage resources utilized by herders-were selected as study subjects. These species differ markedly in morphological structure, palatability, and nutrient composition, making them ideal candidates for comparative evaluation of component-level nutritional value. The CRITIC-GRA-TOPSIS method was applied at the stem-leaf component level to systematically evaluate crude protein (CP), ether extract (EE), crude ash (CA), neutral detergent fiber (NDF) and acid detergent fiber (ADF). This revealed the extent of differences in nutrient content and feeding value between the leaves and stems of these four forage species. Conducting multidimensional nutritional evaluations at the component scale to derive species rankings, and validating model robustness through discrimination tests, provides scientific basis for precise grazing management and high-quality forage selection in desert steppes.

**Figure 1:**
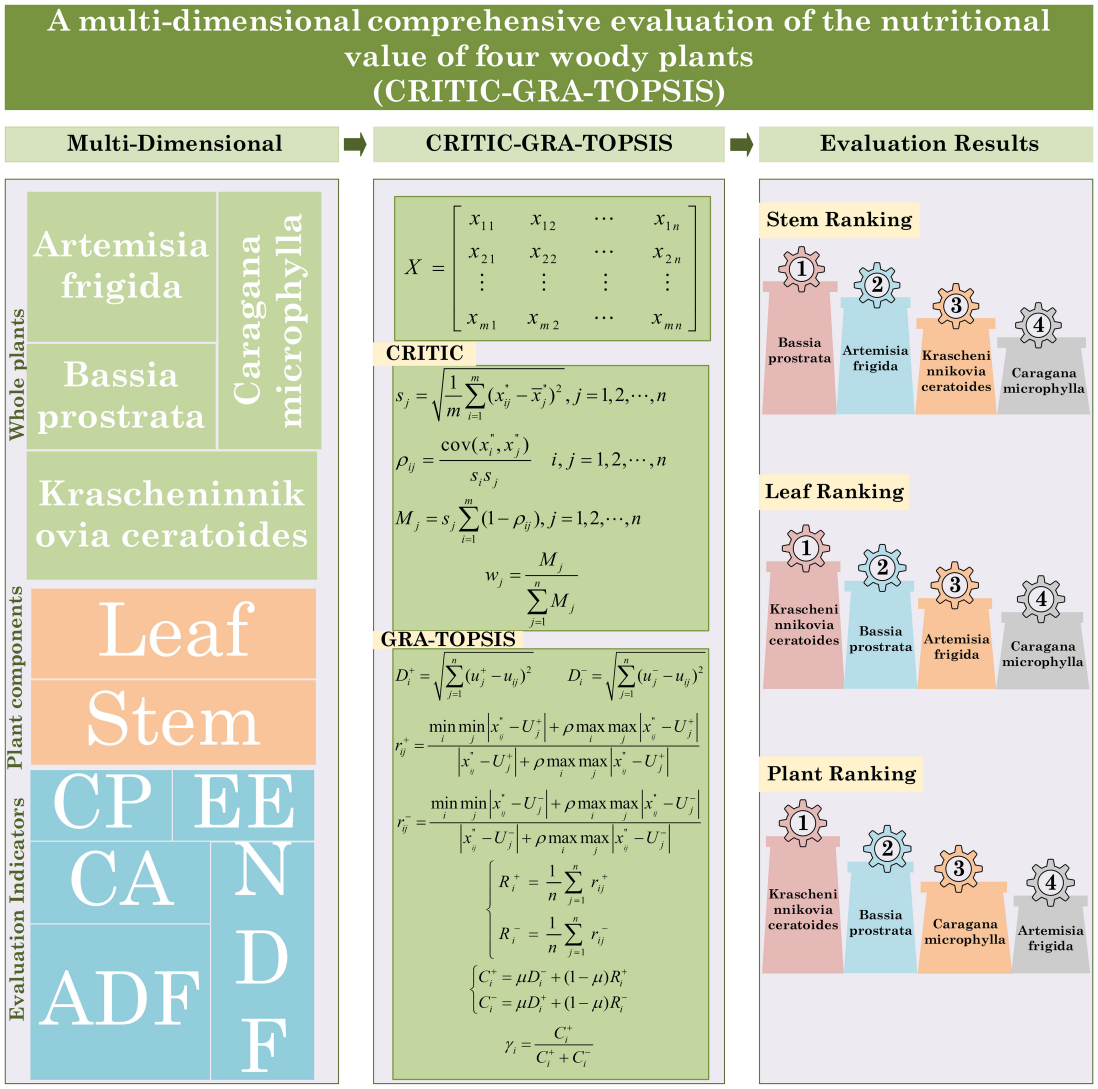
Technical roadmap for the paper.

To present the full content more clearly, create a technical roadmap (**Figure 1**):

## 2 Materials and methods

### 2.1 Study area overview

The experimental pasture sampling site is located in Siziwang Banner, Ulanqab City, Inner Mongolia (Xie et al., 2019) (Geographical coordinates: 41°10′–43°22′N, 110°20′–113°00′E), with an average elevation of 1000–1500 meters. This region lies on the Mongolian Plateau at the northern foothills of the Yin Mountains, characterized primarily by plateau terrain. The southern part consists of the Yin Mountain range, while the northern section transitions into desert steppe. The overall topography exhibits a south-high, north-low gradient. The climate of the entire banner exhibits typical temperate continental characteristics, marked by aridity, low rainfall, abundant sunshine, and significant temperature variations. The annual average temperature ranges from 1 to 6 °C, with annual precipitation averaging 240 to 386 mm, primarily concentrated between June and August. The seasons are distinctly marked: winters are cold and long, summers are brief and cool, springs bring sudden temperature rises and frequent sandstorms, and autumns experience sharp temperature drops with a short frost-free period. Pasture species at the experimental site include *Artemisia frigida*, *Bassia prostrata*, *Krascheninnikovia ceratoides*, and *Caragana microphylla*.

### 2.2 Sampling and indicator measurement

Based on years of survey data, sample plots (1m × 1m) were randomly established within representative pasture areas for each species. Within each plot, three subsamples were collected and measured separately as technical replicates (n=3 per species per component). A minimum of three replicates per species and plant component—the minimum number required for valid ANOVA in vegetation surveys—was adopted to ensure adequate statistical power for detecting differences among species and components, while balancing sampling effort against the logistical constraints of field collection in the remote desert steppe environment. Sampling was conducted in mid-to-late August when forage reached peak flowering and biomass levels, meeting experimental requirements. Manual cutting left stubble at approximately 5 cm, followed by fresh weight measurement for each forage species. Minimum fresh sample collection of 1000 g per species ensured representativeness. Leaves and stems were manually separated. Samples of the same forage species were thoroughly mixed, placed in archival bags, and dried in a 65 °C constant-temperature oven for 48 hours. All dried stem-leaf samples were ground using a pulverizer, sieved through a 1 mm mesh, and subdivided using the quartering method to ensure sample homogeneity (Li et al., 2016).

The measured nutritional components of the forage included CP, EE, CA, ADF, and NDF. CP content was determined using a fully automated Dumas nitrogen analyzer, while EE content was measured with a VELP SER148/6 fat tester. CA content was determined by the 550 °C combustion method. ADF and NDF contents were measured using the Van Soest method (Soest, 1967).

### 2.3 Statistical analysis and evaluation methods

Perform analysis of variance and multiple comparisons using IBM SPSS Statistics 27 to test for significant differences among indicators. Utilize software such as Origin 2025 for data analysis and graph generation.

To investigate whether there are significant differences in the nutritional content of forage across different plant components, the same components in different plants, and the entire plant in multiple dimensions, analysis of variance (ANOVA) and multiple comparisons were conducted using IBM SPSS Statistics 27. This was done to determine whether significant differences existed among the 10 measured indicators, thereby laying a theoretical foundation for subsequent research. Next, CRITIC weighting is applied to each of the three dimensions. The GRA-TOPSIS comprehensive evaluation method is then introduced to rank the nutritional value of forage grasses. Finally, a discrimination test is conducted to validate the evaluation method, ensuring the accuracy of the research.

#### 2.3.1 Analysis of variance and multiple comparison methods

Analysis of variance (ANOVA) (Pantula and Pollock, 1985) is a statistical method used to test whether the differences in means among three or more samples are significant. It is widely applied in fields such as agricultural experiments and the social sciences. The core concept of ANOVA is to decompose total variance into within-group variance and between-group variance. By analyzing the different sources of variation in the data, it determines whether a particular factor or treatment has a significant effect on the results.

Prior to ANOVA, residual diagnostic plots were inspected to verify that all nutritional datasets approximately satisfied the normality and homogeneity of variance assumptions required for valid variance analysis. When ANOVA indicated significant intergroup differences, Tukey’s HSD test was adopted for *post-hoc* multiple comparisons to correct cumulative type I error and accurately locate pairwise differences between groups.

#### 2.3.2 Fundamental principles of the CRITIC-GRA-TOPSIS integrated evaluation method

##### 2.3.2.1 CRITIC empowerment method

Assuming there are *m* alternatives and *n* criteria, construct the original evaluation matrix:


$$X=\left[\begin{array}{cccc} x_{11} & x_{12} & \cdots & x_{1n}\\ x_{21} & x_{22} & \cdots & x_{2n}\\ \vdots & \vdots & \vdots & \vdots \\ x_{m1} & x_{m2} & \cdots & x_{mn} \end{array}\right]$$

1. Indicator direction alignment processing.

For benefit-oriented indicators, higher values indicate better performance; for cost-oriented indicators, lower values indicate better performance. When both types of indicators coexist, data must be standardized to facilitate subsequent analysis.

For benefit-oriented indicators:

(1) $$x_{ij}^{\prime }=\frac{x_{ij}-x_{j\min}}{x_{j\max}-x_{j\min}}$$

For cost-based indicators:

(2) $$x_{ij}^{\prime }=\frac{x_{j\max}-x_{ij}}{x_{j\max}-x_{j\min}}$$

Where *x_j_*_min_ represents the minimum value of the *j*th indicator; *x_j_*_max_ represents the maximum value of the *j*th indicator. After processing using the above formula, the normalized matrix *X*′ is obtained.

2. Dimensionless processing of indicator data.

Since the indicators in matrix *X*′ have different meanings, normalization methods must be applied to each indicator’s data to perform dimensionless processing, yielding the standardized matrix *X*″:

(3) $$x_{ij}^{\prime \prime }=\frac{x_{ij}^{\prime }}{\sqrt{\sum_{j=1}^{n}{x_{ij}^{\prime }}^{2}}},\;i=1,2,\cdots ,m$$

3. Calculate the objective weight of the indicator.

First, calculate the comparison strength, i.e., the standard deviation:

(4) $$s_{j}=\sqrt{\frac{1}{m}\sum_{i=1}^{m}{\left(x_{ij}^{\prime \prime }-\bar{x_{ij}^{\prime \prime }}\right)}^{2}},\;j=1,2,\cdots ,n$$

Next, calculate the correlation coefficient, i.e., the correlation coefficient:

(5) $$\rho_{ij}=\frac{\mathrm{cov}(x_{i}^{\prime \prime },x_{j}^{\prime \prime })}{s_{i}s_{j}}\;i,j=1,2,\cdots ,n$$

Where $\bar{x_{ij}^{\prime \prime }}$ denotes the mean of the *j*th indicator; $\mathrm{cov}\left(x_{i}^{\prime \prime },x_{j}^{\prime \prime }\right)$ represents the covariance between the *i*th and *j*th columns of the standardized matrix *X*″.

Let *M_j_* denote the information content contained in the *j*th evaluation indicator. Then *M_j_* can be expressed as:

(6) $$M_{j}=s_{j}\sum_{i=1}^{m}\left(1-\rho_{ij}\right),j=1,2,\cdots ,n$$

The larger the value of *M_j_*, the greater the information content contained in the *j*th indicator, and the more important that indicator becomes, thus requiring a higher weight. Therefore, the weight calculation formula for the *j*th indicator is:

(7) $$w_{j}=\frac{M_{j}}{\sum_{j=1}^{n}M_{j}}$$

##### 2.3.2.2 GRA-TOPSIS integrated evaluation model

1. Construct the weighted evaluation matrix. Let the normalized matrix *X*″ obtained from the CRITIC method serve as the initial evaluation matrix, and construct the weighted evaluation matrix:

(8) $$\begin{array}{l} u_{ij}=w_{j}\times x_{ij}^{\prime \prime }\\ U=\left[\begin{array}{ccc} u_{11} & \cdots & u_{1n}\\ \vdots & \vdots & \vdots \\ u_{m1} & \cdots & u_{mn} \end{array}\right] \end{array}$$

In the formula, *w_j_* represents the weight of the indicator, $x_{ij}^{\prime \prime }$ denotes the standardized element of the evaluation matrix, *u_ij_* signifies the elements of the weighted matrix, and *U* denotes the weighted matrix.

2. Determine the positive and negative ideal solutions ${U_{j}}^{+}$ and ${U_{j}}^{-}$.

(9) $$U_{j}^{+}=(u_{1}^{+},u_{2}^{+},\cdots ,u_{n}^{+})=\{\max u_{ij}|i=1,2,\cdots ,m\}$$

(10) $$U_{j}^{-}=(u_{1}^{-},u_{2}^{-},\cdots ,u_{n}^{-})=\{\min u_{ij}|i=1,2,\cdots ,m\}$$

3. Calculate the Euclidean distances $D_{i}^{+}$ and $D_{i}^{-}$ between the positive and negative ideal solutions.

(11) $$D_{i}^{+}=\sqrt{\sum_{j=1}^{n}{\left(u_{j}^{+}-u_{ij}\right)}^{2}}$$

(12) $$D_{i}^{-}=\sqrt{\sum_{j=1}^{n}{\left(u_{j}^{-}-u_{ij}\right)}^{2}}$$

4. Calculate the grey relational coefficients $r_{ij}^{+}$ and $r_{ij}^{-}$ for the positive and negative ideal solutions.

(13) $$r_{ij}^{+}=\frac{\underset{i}{\min}\;\underset{j}{\min}\left|x_{ij}^{\prime \prime }-U_{j}^{+}\right|+\rho \;\underset{i}{\max}\;\underset{j}{\max}\left|x_{ij}^{\prime \prime }-U_{j}^{+}\right|}{\left|x_{ij}^{\prime \prime }-U_{j}^{+}\right|+\rho \;\underset{i}{\max}\;\underset{j}{\max}\left|x_{ij}^{\prime \prime }-U_{j}^{+}\right|}$$

(14) $$r_{ij}^{-}=\frac{\underset{i}{\min\;}\underset{j}{\min}\left|x_{ij}^{\prime \prime }-U_{j}^{-}\right|+\rho \;\underset{i}{\max}\;\underset{j}{\max}\left|x_{ij}^{\prime \prime }-U_{j}^{-}\right|}{\left|x_{ij}^{\prime \prime }-U_{j}^{-}\right|+\rho \;\underset{i}{\max}\;\underset{j}{\max}\left|x_{ij}^{\prime \prime }-U_{j}^{-}\right|}$$

Here, *ρ* denotes the resolution coefficient, and generally, *ρ* = 0.5, which is typically set to 0.5 to maximize the differentiation among alternatives (Shou et al., 2023).

5. Calculate the grey correlation coefficients $R_{i}^{+}$ and $R_{i}^{-}$.

(15) $$\{\begin{array}{c} R_{i}^{+}=\frac{1}{n}\sum_{j=1}^{n}r_{ij}^{+}\\ R_{i}^{-}=\frac{1}{n}\sum_{j=1}^{n}r_{ij}^{-} \end{array}$$

(6) Combining Euclidean distance with grey correlation to construct a novel weighted distance metric.

(16) $$\{\begin{array}{c} C_{i}^{+}=\mu D_{i}^{-}+(1-\mu )R_{i}^{+}\\ C_{i}^{-}=\mu D_{i}^{+}+(1-\mu )R_{i}^{-} \end{array}$$

Where *μ* is determined subjectively by the decision-maker. Generally, setting *μ* = 0.5 is adopted to assign equal preference weights to both evaluation methods (Shou et al., 2023).

7. Calculate the new relative proximity measure *γ_i_*.

(17) $$\gamma_{i}=\frac{C_{i}^{+}}{C_{i}^{+}+C_{i}^{-}}$$

##### 2.3.2.3 Discrimination test

Discrimination tests (Wu, 2018) provide a quantitative measure of the discriminant validity of ranking systems. They indicate whether the system can sensitively identify genuine differences among evaluation subjects. Higher values signify greater reliability in the rankings and stronger robustness of the method against interference. This method measures the degree of dispersion and discrimination in ranking results. An evaluation system with high discrimination capacity generates score sequences with greater dispersion, thereby revealing the relative order of evaluation subjects more clearly and reliably. This indicates that the method possesses a low sensitivity threshold (i.e., it can detect finer distinctions). The calculation formula is as follows:

(18) $$D=\frac{\sum_{i=1}^{m-1}\sqrt{{\left(V_{i+1}^{*}-V_{i}^{*}\right)}^{2}+{\left(N_{i+1}-N_{i}\right)}^{2}}}{\sqrt{{\left(V_{m}^{*}-V_{1}^{*}\right)}^{2}+{\left(N_{m}-N_{1}\right)}^{2}}}=\frac{\sum_{i=1}^{m-1}\sqrt{{\left(V_{i+1}^{*}-V_{i}^{*}\right)}^{2}+1}}{\sqrt{2m^{2}-2m-1}}$$

Where *m* denotes the number of evaluation subjects; $V_{i}^{*}=m\times (1-\frac{\left|V_{1}-V_{i}\right|}{V_{1}-V_{m}})(1\leq i\leq m)$ denotes the standardized comprehensive evaluation index for the *i*th evaluation object; *V_i_* and *N_i_* (1 ≤ *i* ≤ *m*) represent the exponent and rank of the *i*th object after sorting by exponent in descending order; If the discrimination index exceeds 1, the composite evaluation index ranking passes the test, indicating that the evaluation results possess a certain degree of stability and reliability (Wu, 2018).

## 3 Results and analysis

### 3.1 Analysis of variance results

#### 3.1.1 Analysis of differences in nutritional value among different forage components within species

**Figure 2:**
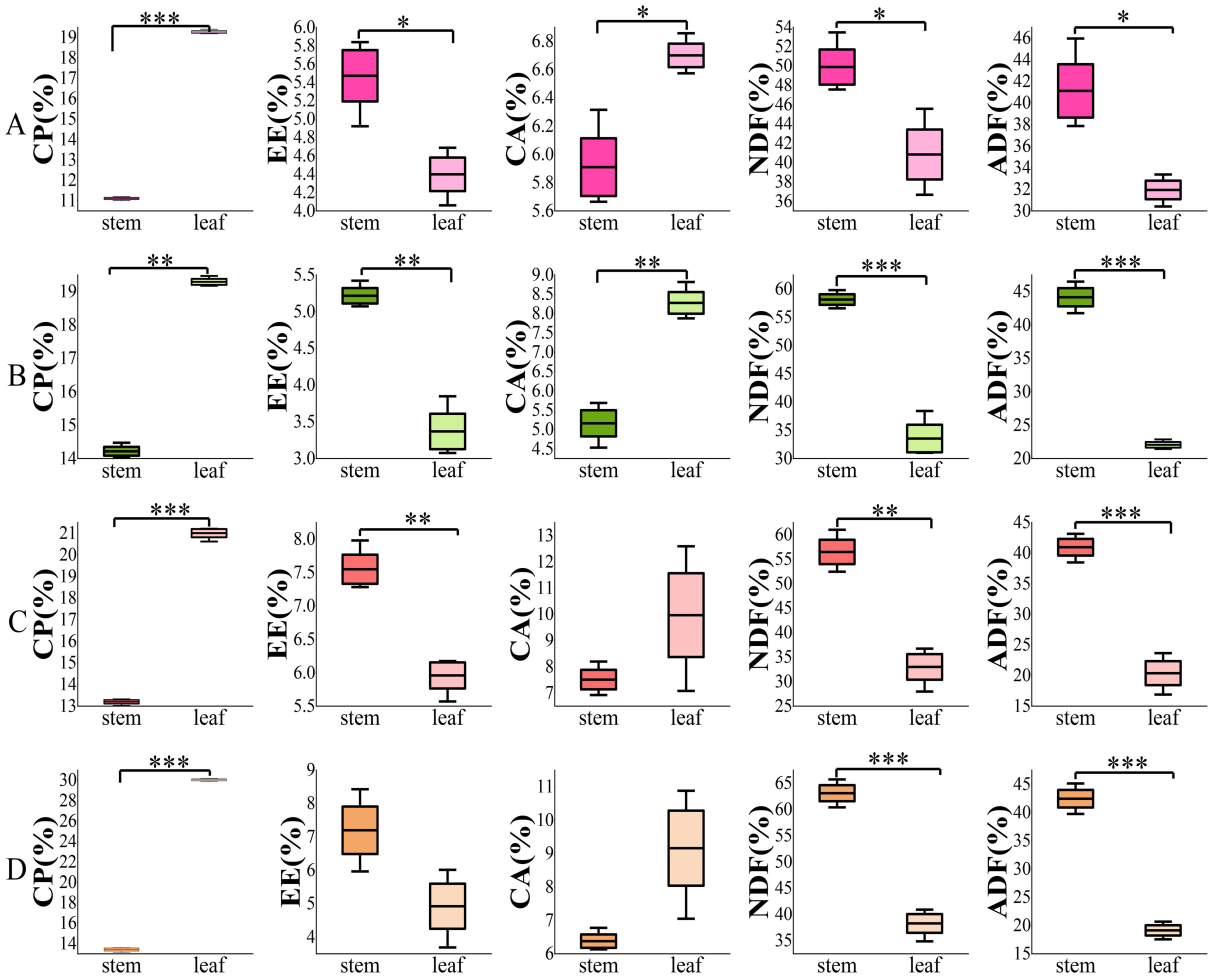
Multivariate boxplot of nutritional indicators for stems and leaves of four forage species. **(A)** *Artemisia frigida*; **(B)** *Caragana microphylla*; **(C)** *Bassia prostrata*; **(D)** *Krascheninnikovia ceratoides*. *P*< 0.05*; *P*< 0.01**; *P*< 0.001***.

To investigate whether there are significant differences in nutrient content between stems and leaves of the same species, ANOVA was employed. The results are presented in **Figure 2**.

Boxplots (**Figure 2**) indicate that leaf CP content was significantly higher than stem CP content across all four species (*P*< 0.01 or *P*< 0.001). *Krascheninnikovia ceratoides* exhibited the highest CP content (approximately 30%), while *Artemisia frigida* had the lowest. In contrast, EE content exhibited the opposite pattern: except for *Krascheninnikovia ceratoides*, where no significant difference was observed between stems and leaves, the EE content in the stems of the other three forage species was significantly higher than in the leaves. CA showed significant differences between *Artemisia frigida* and *Caragana microphylla* (*P*< 0.05), while no significant difference was found between *Bassia prostrata* and *Krascheninnikovia ceratoides*. Regarding fiber indicators, NDF and ADF showed consistent patterns across all four forage species (*P*< 0.05 or *P*< 0.01), with stems exhibiting significantly higher levels than leaves.

#### 3.1.2 Analysis of nutritional value differences in stem among species

**Figure 3:**
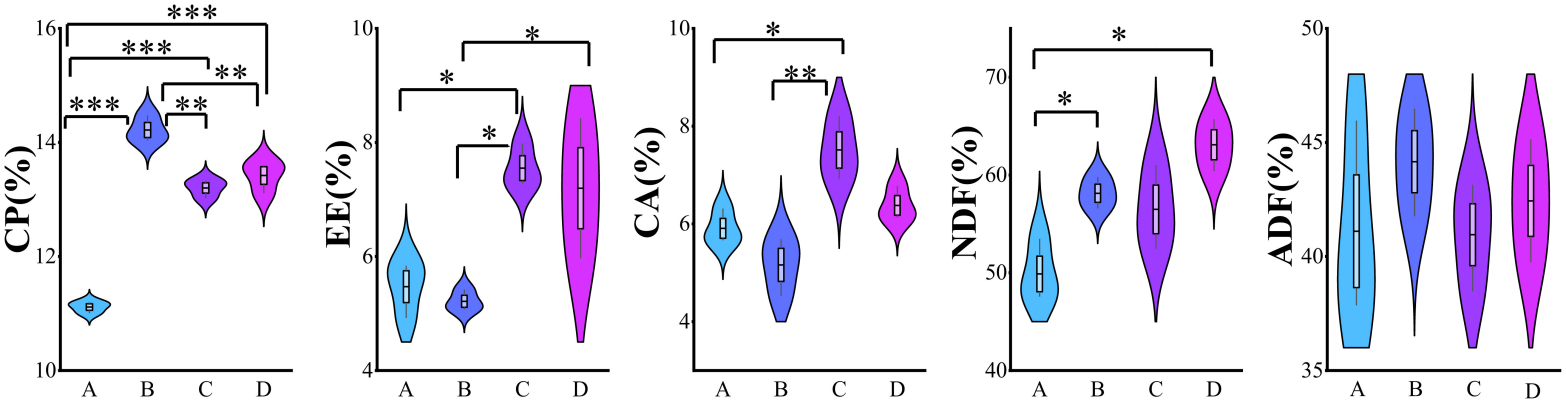
Violin chart of five stem indicators. A: *Artemisia frigida*; B: *Caragana microphylla*; C: *Bassia prostrata*; D: *Krascheninnikovia ceratoides*. *P*< 0.05*; *P*< 0.01**; *P*< 0.001***.

To investigate whether there are significant differences in the nutritional components contained in the stems of different species, a variance analysis was conducted on five indicators. The results are shown in **Figure 3**.

Violin plot (**Figure 3**) analysis of five nutritional indicators in the stems of four forage species reveals distinct species-specific distributions across different nutritional components. In terms of CP content, *Artemisia frigida* exhibited extremely significant differences from the other three forage species (*P*< 0.001). *Caragana microphylla* showed extremely significant differences from both *Bassia prostrata* and *Krascheninnikovia ceratoides* (*P*< 0.001). Significant differences in EE were observed between *Artemisia frigida* and *Bassia prostrata*, between *Caragana microphylla* and *Bassia prostrata*, and between *Caragana microphylla* and *Krascheninnikovia ceratoides* (*P*< 0.05). *Artemisia frigida* and *Bassia prostrata* showed significant differences in CA (*P*< 0.05), while *Caragana microphylla* and *Bassia prostrata* exhibited extremely significant differences in CA (*P*< 0.01). Significant differences were observed between *Artemisia frigida* and *Caragana microphylla*, as well as between *Artemisia frigida* and *Krascheninnikovia ceratoides*, in NDF (*P*< 0.05). No significant differences were found among the four forage species in ADF.

#### 3.1.3 Analysis of nutritional value differences among leaf of different species

**Figure 4:**
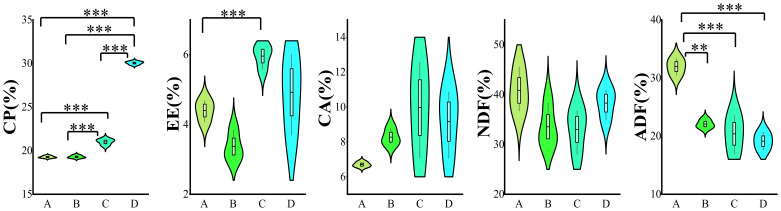
Violin chart of five leaf indicators. A: *Artemisia frigida*; B: *Caragana microphylla*; C: *Bassia prostrata*; D: *Krascheninnikovia ceratoides*. *P*< 0.05*; *P*< 0.01**; *P*< 0.001***.

To investigate whether there are significant differences in nutrient content among leaves of different species, a variance analysis was conducted on five indicators. The results are shown in **Figure 4**.

The violin plot for five nutritional indicators of leaves across different forage species reveals (**Figure 4**) that leaf nutrient composition exhibits greater variation between species. In terms of CP content, *Artemisia frigida* exhibited extremely significant differences (*P*< 0.001) in leaf samples compared to *Bassia prostrata* and *Krascheninnikovia ceratoides*. *Caragana microphylla* showed extremely significant differences from *Bassia prostrata* and *Krascheninnikovia ceratoides* (*P*< 0.001), while *Bassia prostrata* exhibited extremely significant differences from *Krascheninnikovia ceratoides* (*P*< 0.001). *Artemisia frigida* and *Bassia prostrata* showed extremely significant differences in EE (*P*< 0.001). No significant differences were observed among the four forage species for CA and NDF. *Artemisia frigida* and *Caragana microphylla* showed extremely significant differences in ADF (*P*< 0.01). *Artemisia frigida* also exhibited extremely significant differences in ADF compared to *Bassia prostrata* and *Krascheninnikovia ceratoides* (*P*< 0.001).

#### 3.1.4 Analysis of differences in nutritional value among whole-plant forages across species

**Figure 5:**
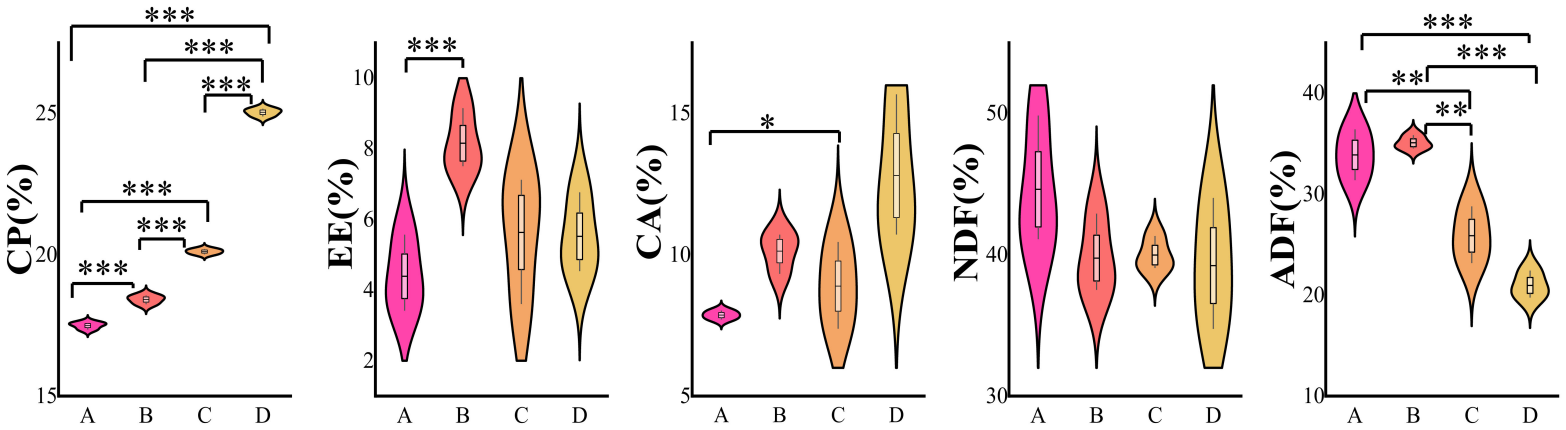
Violin chart of 10 plant indicators. A: *Artemisia frigida*; B: *Caragana microphylla*; C: *Bassia prostrata*; D: *Krascheninnikovia ceratoides*. *P*< 0.05*; *P*< 0.01**; *P*< 0.001***.

To investigate whether there were significant differences in nutrient content among different plant samples, a variance analysis was conducted on five indicators. The results are shown in **Figure 5**.

The overall plant level reflects the combined characteristics of stem and leaf components. The violin plots of the whole plants for the four forage species (**Figure 5**) indicate extremely significant differences (*P*< 0.001) in CP content between each pair of species. *Artemisia frigida* and *Caragana microphylla* showed extremely significant differences (*P*< 0.001) in EE. *Artemisia frigida* and *Bassia prostrata* showed significant differences in CA (*P*< 0.05). No significant differences were observed among all species for NDF. *Artemisia frigida* versus *Bassia prostrata* and *Caragana microphylla* versus *Bassia prostrata* exhibited extremely significant differences in ADF (*P*< 0.01). *Artemisia frigida* and *Krascheninnikovia ceratoides*, as well as *Caragana microphylla* and *Krascheninnikovia ceratoides*, showed highly significant differences in ADF (*P*< 0.001).

### 3.2 CRITIC-GRA-TOPSIS integrated evaluation results

#### 3.2.1 CRITIC weighting results across three dimensions: stem, leaf, and whole plant

Among the nutritional indicators of this forage, components such as cellulose and lignin contained within NDF and ADF are virtually indigestible by animal digestive enzymes, which contributes to diminished nutritional value. Therefore, these two indicators are treated as cost-based metrics, while the remaining ones are considered benefit-based metrics for subsequent analysis.

The weighting results of the CRITIC method (**Table 1**) comprehensively calculated the volume of information for each indicator across two dimensions-Variability and Conflictual-thereby determining their respective weights within the integrated evaluation system (Equations 1–7).

**Table 1:** CRITIC weighting results for stems, leaves, and whole plants.

Component	Indicator	Variability	Conflictual	Volume of information	Weights
Stem	CP	0.301	5.242	1.579	0.192
	EE	0.386	4.724	1.822	0.222
	CA	0.322	5.475	1.760	0.214
	NDF	0.350	4.709	1.649	0.201
	ADF	0.313	4.500	1.409	0.171
Leaf	CP	0.473	3.806	1.799	0.224
	EE	0.338	3.920	1.325	0.165
	CA	0.372	6.667	2.477	0.309
	NDF	0.344	4.162	1.430	0.178
	ADF	0.293	3.389	0.994	0.124
Plant	CP	0.418	3.442	1.439	0.197
	EE	0.386	4.088	1.576	0.216
	CA	0.312	6.568	2.046	0.280
	NDF	0.290	2.940	0.854	0.117
	ADF	0.397	3.516	1.397	0.191

In the stem component evaluation system, EE and CA carry the greatest information content (1.822 and 1.760, respectively), and thus are assigned the highest weights (0.222 and 0.214). The high weights for these two indicators primarily stem from their outstanding comprehensive performance: on one hand, they exhibit high variability (EE: 0.386; CA: 0.322); on the other hand, they exhibit strong conflict (EE: 4.724; CA: 5.475). CP, despite having the lowest variability (0.301), possesses the strongest conflict (5.242), enabling it to maintain relatively high information content (1.579) and weight (0.192). In contrast, fiber-related indicators present a different pattern. Although the variability of NDF and ADF is not low (0.350 and 0.313, respectively), their conflict levels are relatively weak (4.709 and 4.500, respectively). This results in their information content (NDF: 1.649; ADF: 1.409) and final weights (NDF: 0.201; ADF: 0.171) were lower than those of core nutritional indicators.

In the leaf component evaluation system, CA demonstrates a prominent core position, with both its information content (2.477) and weight (0.309) significantly exceeding other indicators. This advantage stems from its outstanding conflict level (6.667) and high variability (0.372). CP exhibits the highest variability among all indicators (0.473). Although its conflict level is relatively low (3.806), its high variability still yields considerable information content (1.799) and weight (0.224), making it the second most important indicator after crude ash. NDF and ADF exhibit acceptable variability (0.344 and 0.293, respectively), but their low conflict scores (4.162 and 3.389) result in lower information content (1.430 and 0.944) and weights (0.178 and 0.124). EE showed no outstanding performance in any aspect of leaf analysis, with variability, conflict, information content, and weight all at moderate or below-average levels.

Within the overall plant component evaluation system, CA remains central, exhibiting the highest conflict (6.568) and maximum information content (2.046), thus receiving the highest weight (0.280). EE and CP demonstrate comparable importance in the overall plant, with weights of 0.216 and 0.197 respectively. EE achieves high information content (1.576) due to its favorable variability (0.386) and moderate conflict (4.088). CP, despite having the lowest conflict (3.442), maintains considerable information content (1.439) owing to its relatively high variability (0.418). ADF exhibited high variability (0.397) but was constrained by moderate conflict (3.516), resulting in moderate information content (1.397) and a weight (0.191) slightly below core nutritional indicators. NDF ranked last in variability, conflict, information content, and weight.

#### 3.2.2 CRITIC-GRA-TOPSIS integrated evaluation of forage nutritional value

To overcome the limitations of single evaluation indicators, this study employed the CRITIC-GRA-TOPSIS integrated evaluation method to comprehensively assess the stems, leaves, and entire plants of four forage species.

This method calculates the Euclidean distance between each scheme and the positive ideal solution ( $D_{i}^{+}$) and negative ideal solution ( $D_{i}^{-}$), as well as their grey correlation degree with the positive ideal solution ( $R_{i}^{+}$) and negative ideal solution ( $R_{i}^{-}$). It then combines these two metrics through weighting to compute the weighted distances $C_{i}^{+}$ and $C_{i}^{-}$, ultimately yielding the relative proximity scores (γ*_i_*) and performing a ranking (Equations 8–17).

The comprehensive evaluation ranking for stems is (**Table 2**): *Bassia prostrata* (0.5597) > *Artemisia frigida* (0.5235) > *Krascheninnikovia ceratoides* (0.4999) > *Caragana microphylla* (0.4925). The comprehensive evaluation ranking of leaves is: *Krascheninnikovia ceratoides* (0.5455) > *Bassia prostrata* (0.4806) > *Artemisia frigida* (0.4528) > *Caragana microphylla* (0.4472).The overall comprehensive evaluation ranking of plant species is: *Krascheninnikovia ceratoides* (0.5482) > *Bassia prostrata* (0.5363) > *Caragana microphylla* (0.4963) > *Artemisia frigida* (0.3893).

**Table 2:** Comprehensive evaluation ranking results for stem, leaf, and whole plant CRITIC-GRA-TOPSIS.

Component	Category	$D_{i}^{+}$	$D_{i}^{-}$	$R_{i}^{+}$	$R_{i}^{-}$	$C_{i}^{+}$	$C_{i}^{-}$	γ*_i_*	Rankings
Stem	*Artemisia frigida*	0.2089	0.2323	0.6611	0.6043	0.4467	0.4066	0.5235	2
	*Bassia prostrata*	0.1903	0.2394	0.7000	0.5483	0.4695	0.3693	0.5597	1
	*Krascheninnikovia ceratoides*	0.2022	0.2019	0.5852	0.5851	0.3936	0.3937	0.4999	3
	*Caragana microphylla*	0.2303	0.2225	0.6408	0.6594	0.4316	0.4449	0.4925	4
Leaf	*Artemisia frigida*	0.2792	0.2730	0.6281	0.8097	0.4505	0.5444	0.4528	3
	*Bassia prostrata*	0.3259	0.2012	0.7394	0.6906	0.4703	0.5082	0.4806	2
	*Krascheninnikovia ceratoides*	0.2254	0.2605	0.7447	0.6123	0.5026	0.4188	0.5455	1
	*Caragana microphylla*	0.2894	0.1923	0.6412	0.7407	0.4167	0.5151	0.4472	4
Plant	*Artemisia frigida*	0.3155	0.2024	0.5363	0.8430	0.3693	0.5793	0.3893	4
	*Bassia prostrata*	0.1922	0.2207	0.6299	0.5431	0.4253	0.3677	0.5363	2
	*Krascheninnikovia ceratoides*	0.2444	0.2616	0.7513	0.5903	0.5064	0.4174	0.5482	1
	*Caragana microphylla*	0.2458	0.2357	0.6453	0.6483	0.4405	0.4470	0.4963	3

### 3.3 Strength analysis results

The biplot (dual-standard plot) was originally proposed by Gabriel (1971), whose core concept involves visualizing the results of bivariate data tables through centralized processing and eigenvalue decomposition. This diagram intuitively reveals associations between rows (e.g., samples) and columns (e.g., variables), thereby facilitating interpretation and inference of the data’s internal structure. The eigenvalue and goodness-of-fit are key indicators for evaluating whether a biplot adequately reveals the inherent structure within the data. An eigenvalue greater than 1 indicates that the principal component contains significant regularity information. Goodness-of-fit refers to the cumulative percentage of total variance explained by the first two principal components. When this value exceeds 70%, it indicates that the biplot can reasonably approximate the latent relationships between variables and samples, meaning it passes the goodness-of-fit test and yields reliable interpretable results (Gabriel, 1971; Wu, 2018).

To visually present the comprehensive evaluation results of the nutritional value of woody pasture plants in Siziwang Banner, Inner Mongolia, and explain the nutritional characteristics of each woody plant species, this study employs the biplot method to analyze the relationships between nutritional indicators and pasture plant types.

**Figure 6:**
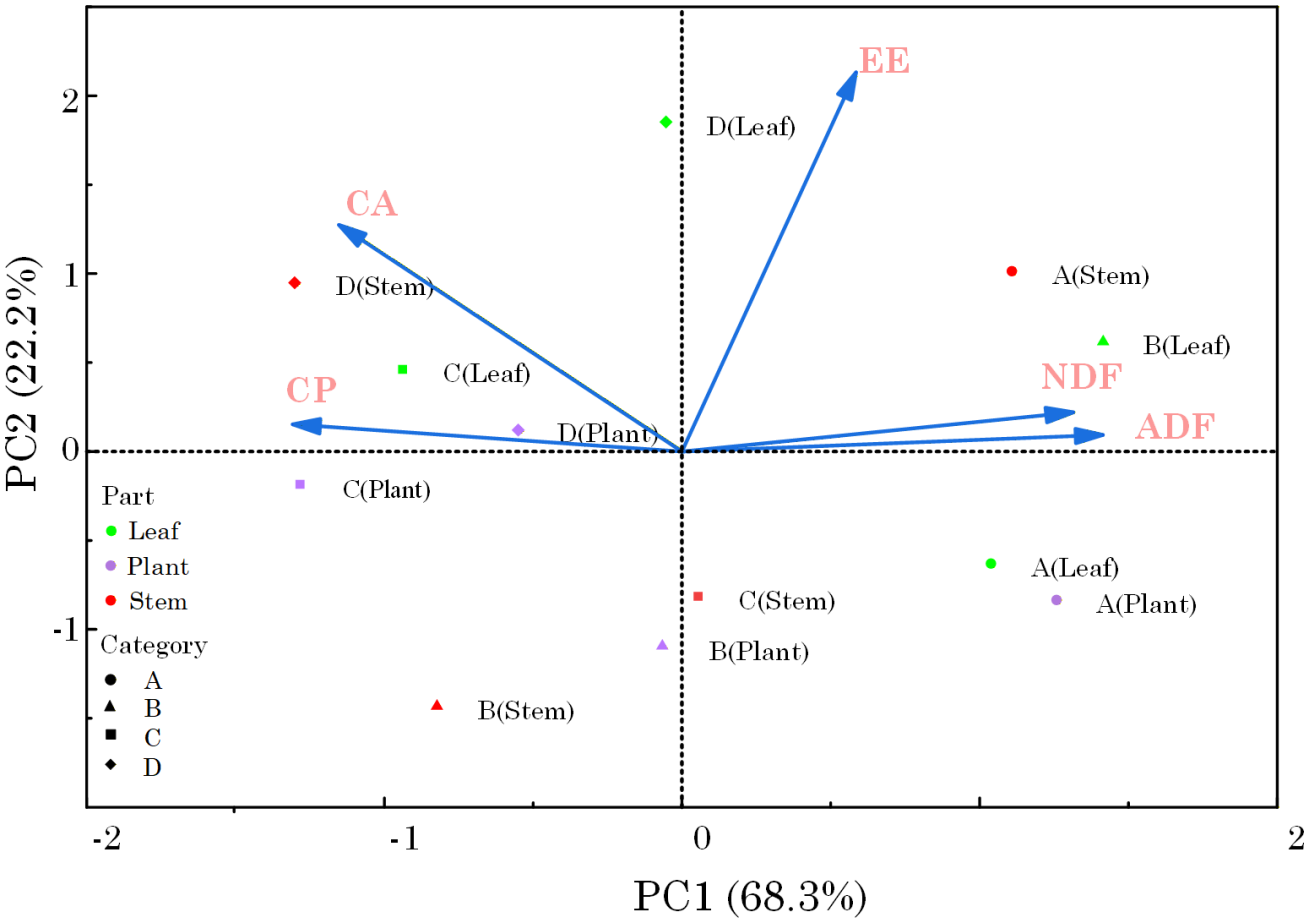
Advantage analysis chart for 5 indicators of four forage categories. A: *Artemisia frigida*; B: *Caragana microphylla*; C: *Bassia prostrata*; D: *Krascheninnikovia ceratoides*.

Based on the analysis of biplot advantages (**Figure 6**), it is evident that the first principal component (PC1) explains 68.3% of the total variance, while the vertical axis (PC2) represents the secondary principal component, explaining 22.2% of the total variance. Together, they account for over 90% of the cumulative variance, with the eigenvalue of PC1 exceeding 1. This indicates the presence of significant nutritional structure information within the biplot. Simultaneously, the cumulative fit between PC1 and PC2 exceeds the 70% threshold, indicating that the biplot effectively reflects the relationship between nutritional indicators and plant species. Taken together, these two criteria—eigenvalue > 1 for PC1 and cumulative variance > 70%—confirm that the biplot provides a reliable representation of the nutritional relationships among species.

As shown in the figure, CP, EE, and CA exhibit a positive correlation, while NDF and ADF also show a positive correlation. Conversely, CP, EE, and CA demonstrate a negative correlation with both NDF and ADF. *Krascheninnikovia ceratoides* and *Bassia prostrata* leaves, along with their whole plants, demonstrate superior crude protein content, ranking first and second respectively. Conversely, *Caragana microphylla* and *Artemisia frigida* leaves, along with their corresponding whole plants, exhibit higher cellulose content and lower nutritional value, placing them last. Regarding the stem dimension, nutritional value correlates with fiber content. *Artemisia frigida* and *Bassia prostrata* rank higher than *Krascheninnikovia ceratoides* and *Caragana microphylla* due to their relatively higher stem cellulose content, while *Krascheninnikovia ceratoides* and *Caragana microphylla* rank lower. The dual-scale diagram provides strong support for the ranking results.

### 3.4 Discrimination test of the CRITIC-GRA-TOPSIS integrated evaluation method

To assess the stability of the comprehensive evaluation method, a discrimination test was employed to evaluate the discriminant validity of the CRITIC-GRA-TOPSIS integrated evaluation system.

As shown in **Table 3**, the discrimination values for stems, leaves, and whole plants were 1.082, 1.118, and 1.113, respectively. All three exceeded the threshold standard of 1. The high discrimination values indicate that this comprehensive evaluation method possesses strong resistance to interference, yielding highly dispersed ranking results. It can clearly and reliably distinguish the true nutritional value differences among the four forage species, thereby ensuring the scientific validity and robustness of the research conclusions (Equation 18).

**Table 3:** Discrimination test results for the CRITIC-GRA-TOPSIS integrated evaluation method.

Component	Stem	Leaf	Plant
Discrimination	1.082	1.118	1.113

## 4 Discussion

This study focused on four dominant woody plants in the desert steppe of Siziwang Banner, Inner Mongolia. The CRITIC-GRA-TOPSIS integrated evaluation method was applied at the stem-leaf component level to systematically compare five nutritional and forage value indicators among these plants, while also validating the robustness of the evaluation method. The results not only reveal significant variations in nutritional quality among different species and plant parts but also provide scientific basis for precision grazing management in desert steppe.

### 4.1 Analysis of variance and multiple comparison results

This study systematically revealed the nutritional variation patterns among four dominant woody plants in desert steppe at the “stem-leaf” scale through analysis of variance and multiple comparisons. Specifically, it investigated differences in quality indicators between different plant components (stem and leaf) within the same forage species, between the same component across different forage species, and between these components and the whole plant. Results indicate that nutritional indicator contents across these three dimensions mostly exhibit significant differences. As shown in **Figure 2**, the leaf components of *Artemisia frigida*, *Bassia prostrata*, *Krascheninnikovia ceratoides*, and *Caragana microphylla* exhibited significantly higher CP and CA than their stems, while stem components NDF and ADF were significantly higher than those in leaves (**Figure 2**). This aligns with findings from *alfalfa* (Milić et al., 2011), *Broussonetia papyrifera* (Hao et al., 2021), and *Sunn* (Lepcha and Naumann, 2021). Edvan and Bezerra (2018) also detailed the differences in nutrient content between stems and leaves in their book. This demonstrates that the widespread superiority of leaves over stems is not coincidental, but rather an inevitable outcome of the long-term coupling between plant functional organ differentiation and diverse environmental conditions. Leaves serve as the center of photosynthesis and assimilation, with their palisade and spongy tissues occupying over 70% of the leaf volume (Borsuk et al., 2022). Chloroplasts are densely distributed, and soluble proteins such as Rubisco and ATP synthase are significantly more abundant than in lignified tissues, forming the physiological basis for a “high-protein, high-metabolism” state with rich cellular contents (Wu et al., 2022). The stem, however, focuses on mechanical support and material transport, with highly lignified vascular bundles and fiber cells (Asaoka et al., 2024). Cell wall thickness reaches 2–4 times that of leaf mesophyll, leading to a sharp increase in structural carbohydrates. This dilutes digestible constituents, resulting in significantly elevated NDF and ADF levels.

Notably, the CP content in *Krascheninnikovia ceratoides* leaves reached 30.05%, far exceeding the prime-grade threshold of CP ≥ 19% for legume hay issued by the American Forage and Grassland Council (Rohweder et al., 1978). This value approaches the level of high-quality leguminous forage grasses (Shah et al., 2020), highlighting its efficient nitrogen accumulation capacity in nutrient-poor habitats and demonstrating significant potential as a high-protein supplementary feed. Leaves CP values of *Bassia prostrata* (21.01%), *Caragana microphylla* (19.29%), and *Artemisia frigida* (19.27%) all surpassed this threshold, with the latter two showing comparable values just above the lower limit of the prime-grade standard. In contrast, stem CP values across the four species fell markedly below the same benchmark: *Caragana microphylla* (14.22%) ranked highest among stems but remained below the 19% threshold; *Bassia prostrata* (13.20%) and *Krascheninnikovia ceratoides* (13.42%) showed moderate levels; and *Artemisia frigida* (11.11%) was substantially lower. Furthermore, stem NDF levels across all four species exceeded the 50% digestibility benchmark (Rohweder et al., 1978), with *Krascheninnikovia ceratoides* showing the poorest digestibility (NDF 63.10%, ADF 42.44%)—consistent with its role as a “supportive stem” adapted to mechanical stress. Analysis of stems (**Figure 3**) and leaves (**Figure 4**) revealed substantial variations in nutritional value among different plant parts of the same species. For stems, *Artemisia frigida* had the lowest CP content, while *Caragana microphylla* had the highest. Conversely, *Krascheninnikovia ceratoides* stems contained high fiber content and low nutritional value. For leaves, both *Bassia prostrata*, *Krascheninnikovia ceratoides* significantly outperformed *Artemisia frigida* and *Caragana microphylla* in CP. Prioritizing retention or artificial reseeding of these two species in grazing areas can enhance the overall nutritional supply capacity of grasslands. **Figure 5** show that *Krascheninnikovia ceratoides* exhibited the highest CP value among all species, followed by *Bassia prostrata*, while *Artemisia frigida* had the lowest values. These results support prioritizing *Krascheninnikovia ceratoides* and *Bassia prostrata* as high-quality forage resources for conservation and cultivation in desert steppe regions, particularly for reseeding degraded pastures. *Artemisia frigida* exhibits low whole-plant nutritional value and is unsuitable as a primary forage. However, due to its strong grazing tolerance, it can serve as an ecological barrier species within pastures.

### 4.2 CRITIC-GRA-TOPSIS integrated evaluation and advantage analysis

In terms of stem quality, the comprehensive ranking of the four forage species is: *Bassia prostrata* >*Artemisia frigida* > *Krascheninnikovia ceratoides*> *Caragana microphylla*. *Bassia prostrata* exhibits significantly high EE and CA in its stems, indicating efficient storage of energy and minerals within the stem tissue, forming a “high-energy stem” type. Despite its relatively high fiber content, it maintains a high overall nutrient density, serving as an important energy supplement source, particularly during cold seasons. Ranked second, *Artemisia frigida* represents the “soft-stem” type. Although it has the lowest crude protein (CP) among the four species, it also has the lowest fiber content and minimal lignification (Gu et al., 2017). Its stems are relatively soft and pliable, making them easily digestible by ruminants. *Krascheninnikovia ceratoides* exhibits extremely high stem fiber content, characteristic of a “supportive stem”. Its high lignification results in poor palatability and digestibility, leading domestic animals to typically consume only the leaves. As a legume, *Caragana microphylla* possesses relatively high CP content but low EE content, resulting in insufficient energy supply. Its elevated fiber content further diminishes its overall value.

For the comprehensive nutritional evaluation and analysis of forage, in terms of leaf and plant dimensions, the comprehensive ranking results for the four forage grasses are as follows: *Krascheninnikovia ceratoides* > *Bassia prostrata*> *Artemisia frigida*> *Caragana microphylla* and *Krascheninnikovia ceratoides* > *Bassia prostrata* > *Caragana microphylla* > *Artemisia frigida*. Leaves serve as the primary site for photosynthesis and the synthesis of carbohydrates and proteins in forage plants (Tcherkez et al., 2020). A higher number of leaves in forage grasses indicates greater nutrient content, signifying higher nutritional value, digestibility, and palatability for the entire plant (Kalu et al., 1990). *Krascheninnikovia ceratoides*, with its deep roots and rosette-like leaf structure, exhibits significantly higher leaf CP (30.05%) than other species in poor habitats. This forage species (Zhao et al., 2012) exhibits well-developed root systems, robust palisade tissue in leaves, and dense chloroplast distribution, conferring physiological traits of drought and cold tolerance. Its relatively high nutritional value, including CP and non-nitrogenous extractives, This high-protein trait likely stems from its strong nitrogen uptake and assimilation capacity, enabling it to maintain high nutritional levels even in barren desert soils. Consequently, it has become a high-yielding, superior forage grass, validating this study’s conclusion that it ranks first in nutritional value. Su et al. (2024) conducted a comprehensive study on three ecotypes of *Krascheninnikovia ceratoides* in Inner Mongolia, indicating that this forage plant possesses high nutritional value, consistent with the conclusions of this paper. Compared to grasses, *Bassia prostrata* has higher crude protein (CP) content, approaching that of legumes. It serves as an important companion or subdominant species in desert steppe (Zhu, 2005), exhibiting “balanced” characteristics. This provides theoretical support for its second-highest composite score among leaves and plants. *Artemisia frigida* leaves are highly nutritious, but its stems have low nutritional value, making it a classic example of a plant with superior leaves and inferior stems. In contrast, *Caragana microphylla* is a plant with balanced nutritional value in both stems and leaves, resulting in a more stable overall nutritional profile. Therefore, the ranking shows that *Artemisia frigida* has slightly higher nutritional value than *Caragana microphylla* at the leaf level, while *Caragana microphylla* has slightly higher nutritional value than *Artemisia frigida* at the plant level.

## 5 Conclusion and recommendations

### 5.1 Conclusion

This study evaluated the nutritional value of four dominant woody species (*Artemisia frigida*, *Bassia prostrata*, *Krascheninnikovia ceratoides*, and *Caragana microphylla*) in the Inner Mongolia desert steppe at the stem-leaf component level using the CRITIC-GRA-TOPSIS integrated evaluation method. The results demonstrate that component-level evaluation reveals substantial nutritional differences masked by whole-plant analysis: leaves consistently exhibited significantly higher CP and lower fiber than stems across all four species, confirming that whole-plant sampling underestimates leaf nutritional value while overestimating stem quality. Notably, *Krascheninnikovia ceratoides* leaves achieved CP content (30.05%) comparable to high-quality leguminous forages, while CP values of all four species’ stems fell below maintenance requirements for grazing ruminants, highlighting the necessity of component-specific nutritional assessment.

Based on comprehensive rankings, *Krascheninnikovia ceratoides* and *Bassia prostrata* are identified as the top-performing forage species, with *Krascheninnikovia ceratoides* ranking first in leaf and whole-plant dimensions, and *Bassia prostrata* first in stem quality. These two species should be prioritized for conservation and reseeding in degraded pastures. In contrast, *Artemisia frigida* showed the lowest whole-plant nutritional value, yet its strong grazing tolerance supports its role as an ecological barrier rather than a primary forage. These findings provide a scientific basis for precision grazing management and forage selection in desert steppe ecosystems, with practical implications for winter supplementary feeding strategies and sustainable grassland utilization.

### 5.2 Recommendations

Based on the component-level nutritional rankings, this study identifies *Krascheninnikovia ceratoides* and *Bassia prostrata* as the top-performing forage species. These two species should be prioritized in grazing management: *Bassia prostrata*, with its exceptionally high leaf CP, serves as an ideal high-protein supplement for livestock during early spring when protein demand is elevated; *Bassia prostrata*, ranking first in stem quality, provides a reliable energy source during cold-season supplementary feeding. In contrast, *Artemisia frigida* and *Caragana microphylla* are not recommended as primary forages due to their lower whole-plant nutritional value. However, *Artemisia frigida* may be retained as an ecological barrier species owing to its strong grazing tolerance.

Furthermore, these findings fill a critical gap in desert steppe forage evaluation: previous studies have predominantly treated whole plants as the analytical unit, thereby underestimating leaf nutritional value and overestimating stem quality. By disaggregating stems and leaves, this study provides a more accurate basis for species-specific utilization and offers a replicable methodological framework for nutritional evaluation of woody forages in other arid and semi-arid regions.

## Data Availability

The original contributions presented in the study are included in the article/supplementary material. Further inquiries can be directed to the corresponding author.
